# Transactions of the American Institute of Homœopathy

**Published:** 1895-12

**Authors:** 


### Transactions of the Forty-seventh Session of the American Institute of Homœopathy. Fiftieth anniversary held at Denver, Col., June 14th to 20th, 1894. Edited under the direction of the Committee of Publication by Pemberton Dudley, M. D., General Secretary. Philadelphia: Sherman & Co., Printers, Seventh and Cherry Streets, 1894.

This large volume was issued at the beginning of the year, and should have been noticed in these pages at that time. The press of other affairs has delayed the notice until now. It would be impossible to give a proper review of the book, and so no more than a notice of it can be attempted. It is graced with a poem by Dr. William Tod Helmuth, of New York, the poet surgeon of the homæopathic school, and one also by Dr. T. P. Wilson, of Cleveland. Of the papers it is impossible to say anything because of the number and extent of them. The best way to gain an insight into so extensive a work is to procure a copy from the General Secretary.

